# Supplementary material for: WordCommentsAnalyzer: A windows software tool for qualitative research
Source: F1000Res. 2018 Sep 4;7:536. Originally published 2018 May 3. [Version 2] doi: 10.12688/f1000research.14819.2 (PMC6137411; doi:10.12688/f1000research.14819.2)
Supplement: Supplementary file 3 [file f1000research-7-17614-s0002.tgz › b2a79496-4ada-47c4-9682-a99ce867836d.zip!/Eight Iranian Health Journals/Journal of Health System Research _34.docx]

© Journal of Health System Research . This work is licensed under CC BY-NC 4.0 (https://creativecommons.org/licenses/by-nc/4.0/).

Volume & Issue: دوره 8، شماره 5؛1391

Title: دیدگاه دانشجویان نسبت به سالمندان

http://hsr.mui.ac.ir/index.php/jhsr/article/view/556

یکی از مشکلات اصلی سالمندان ناتوانی است. بخش زیادی از این ناتوانی ناشی از افرادی است که به سالمندان بر چسب ناتوان بودن می‌زنند. این عامل موجب می‌شود که سالمندان به این باور برسند که ناتوان هستند و همان طور که فکر می‌کنند، عمل می‌کنند (ناتوان، نیازمند به کمک دیگران، کم انرژی و ...) (1). از طرفی حدود یک سوم سالمندان قادر به انجام فعالیت‌های روزمره نیستند (2). 30 درصد سالمندان معتقدند که زندگی معنی خود را برای آن‌ها از دست داده است (3). بخشی از این عامل ناشی از القای دیگران و خودالقایی است. از این رو دیدگاه اطرافیان سالمند از اهمیت بسیار زیادی برخوردار است. از سوی دیگر اهمیت مسأله سالمندان به حدی است که شعار سال 2012 سازمان جهانی بهداشت نیز به موضوع سالمندان اختصاص یافته است. بنابراین مطالعه حاضر با هدف تعیین دیدگاه دانشجویان نسبت به سالمندی طراحی و اجرا شد. این مطالعه از نوع کیفی است که در سال 1391 انجام شد. در این مطالعه، 16 نفر از دانشجویان کارشناسی بهداشت عمومی به روش نمونه‌گیری تصادفی انتخاب شدند. اطلاعات از طریق بحث گروهی متمرکز جمع‌آوری شد. همچنین از بحث‌ها یادداشت برداری شد. از روش تحلیل محتوا جهت تجزیه و تحلیل داده‌ها استفاده شد. تحلیل یادداشت‌ها منجر به استخراج دو دسته ویژگی اصلی از دیدگاه دانشجویان نسبت به سالمندی شد؛ به طوری که سالمندان از دیدگاه دانشجویان دارای 10 ویژگی منفی و 5 ویژگی مثبت بودند. ویژگی‌های منفی شامل زودرنجی، بی‌حوصلگی، تنهایی، افسردگی، مظلوم بودن، فارغ از کار و فعالیت، نیازمند مراقبت، پرخاشگری، در معرض بیماری بودن و ناتوانی بود. ویژگی‌های مثبت سالمندان از دیدگاه دانشجویان عبارت از با ایمان بودن، مهربانی، پشتیبان بودن، با تجربه بودن و اقتدار بود.نتایج نشان داد که دیدگاه دانشجویان نسبت به سالمندان در سطح مطلوبی نیست و دانشجویان ویژگی‌های منفی زیادی را به سالمندان نسبت ‌دادند. از این رو پیشنهاد می‌شود که اقدامات مقتضی به ویژه مداخلات آموزشی جهت اصلاح نگرش دانشجویان و سایر افراد جامعه صورت گیرد. از این طریق می‌توان میزان ناتوانی ناشی از القا به سالمندان را کاهش داد.

Title: بررسی ارتباط تغذیه و سرطان معده: مروری بر شواهد اپیدمیولوژیک

http://hsr.mui.ac.ir/index.php/jhsr/article/view/329

مقدمه: سرطان معده، چهارمین سرطان شایع دنیاست و رتبه دوم مرگ و میر ناشی از سرطان را به خود اختصاص داده است. اگر چه عفونت هلیکوباکتر پیلوری یک عامل بسیار مهم در ابتلا به سرطان معده است، اما تنها عامل خطرزای ایجاد بیماری به شمار نمی‌آید و نقش تغذیه به عنوان مهم‌ترین عامل محیطی در بروز، کنترل و پیش‌گیری از سرطان معده، بسیار مورد مطالعه قرار گرفته است. مطالعه حاضر با هدف، مروری بر مطالعات پیشین جهت بررسی ارتباط بین چندین عامل رژیمی و سرطان معده انجام شد. روش‌ها: جستجو در PubMed با کلید واژه‌های Gastric cancer، Helicobacter pylori،Fruit and vegetables، Meat and processed meat، Salt and salted food، Green tea، Black tea و Coffee صورت گرفت. 36 مطالعه با طراحی‌های اکولوژیک، مقطعی، مورد- شاهدی، کوهرت (Cohort) و متاآنالیز انتخاب شدند و نتایج مطالعات انجام شده در این زمینه تا سال 2011 مورد بررسی قرار گرفت. یافته‌ها: خطر سرطان معده با مصرف بالای میوه‌ها و سبزیجات تازه و شاید چای سبز کاهش ‌یابد، مصرف انواع گوشت‌های فرآوری شده، نمک و غذاهای نمک سود شده، با افزایش خطر سرطان معده همراه است و شواهد روشنی مبتنی بر چگونگی ارتباط بین انواع گوشت‌ها، ماهی، چای سیاه، قهوه و سرطان معده موجود نیست. نتیجه‌گیری: تعدیل رژیم غذایی به سمت افزایش مصرف میوه‌ها و سبزیجات تازه و کاهش مصرف نمک، غذاهای نمک سود شده و گوشت‌های فرآوری شده می‌تواند یک استراتژی بسیار مهم در کنترل و پیش‌گیری از سرطان معده باشد.

Title: گروه‌های غذایی مصرفی و سرطان سینه: مروری بر شواهد موجود

http://hsr.mui.ac.ir/index.php/jhsr/article/view/473

مقدمه: يکي از عوامل تأثيرگذار بر افزايش یا کاهش خطرات سرطان سینه، الگوهای غذایی مختلف متشکل از گروه‌های غذایی متفاوت است. بنابراين نقش رژیم غذایی در این میان، مورد توجه واقع شده است؛ به طوری که مطالعات گسترده‌ای تأثیر گروه‌های غذایی بر خطر سرطان سینه را مورد بررسی قرار داده‌اند. هدف از انجام مطالعه حاضر، مروري بر مطالعات جديد انجام شده در زمينه ارتباط مصرف گروه‌های غذایی مختلف با خطر سرطان سینه بود. مواد و روش‌ها: برای دستیابی به مطالعات موجود در زمینه موضوع مورد بررسی، جستجو بین سال‌های 2009 تا 2011 جهت بررسی مقالات چاپ شده در سال‌های جدید، با عبارات کلیدی Dietary fats، Meat and prooced meat، Fruit and vegetable، Food group، Carbohydrate و Grains و ارتباط آن با سرطان سینه در بانک اطلاعاتی PubMed صورت گرفت. جستجوی این واژه‌ها فقط در عنوان و خلاصه مقاله محدود گشت و تنها در مطالعات انسانی استفاده شد که حدود 400 مقاله مشخص گردید و پس از حذف مقاله‌های بدون ارتباط با موضوع، در نهایت 31 مطالعه مرتبط که متن آن‌ها در دسترس بود، برای مرور در مطالعه حاضر مورد بررسی قرار گرفت. يافته‌ها: برخي مطالعات نشان داده‌اند که مصرف الگوی غذایی غنی از غذاهای گیاهی و استفاده کمتر از گوشت‌ها و مواد غذایی فراوری شده با کاهش خطر سرطان سینه مرتبط است؛ در حالی که مطالعات ديگر به چنين نتيجه‌اي دست نيافته بودند. همچنين تحقيقات، مصرف گروه سبزیجات و میوه‌ها خاصی را به عنوان عامل اثرگذارتري نسبت به کل گروه سبزیجات بر کاهش خطر سرطان سینه عنوان مي‌کنند و ارتباط مصرف گوشت‌های فراوری شده با افزایش خطر سرطان سینه به صورت همسو در مطالعات نشان داده شده بود. نتيجه‌گيري: از آن‌جايي که يافته‌هاي حاصل از مطالعات در زمينه تأثير مصرف الگوی غذایی غنی از سبزیجات دارای عوامل آنتی‌اکسیدانی و کربوهیدرات‌های با شاخص گلایسمیک (Glycemic index یا GI) پایین بر کاهش خطر سرطان سینه همسو می‌باشد، به نظر مي‌رسد که بيشتر مطالعات آينده در اين خصوص بايد ارتباط الگوهای غذایی مختلف را با توجه به عوامل هورمونی و ژنتیکی افراد مورد آزمون قرار دهند.

Title: بررسي غلظت فلزات سنگين روي، سرب، کروم و کادميوم در آب و رسوب خليج گرگان و مصب رودخانه گرگانرود

http://hsr.mui.ac.ir/index.php/jhsr/article/view/288

مقدمه: فلزات سنگين شامل دو دسته عناصر ضروري و غير ضروري هستند که در بوم سم‌شناسي قابل توجه هستند. اين عناصر پايداري بالايي دارند و توانايي ايجاد سميت در موجودات زنده را دارند. هدف از اين پژوهش پایش غلظت فلزات سنگين در آب و رسوب خلیج گرگان و مصب رودخانه گرگانرود می‌باشد. امید است که نتایج به دست آمده برای مقایسه مقادیر در مطالعه‌های آینده به عنوان مبنایی جهت تعیین روند تغییرات مدنظر قرار گیرد. روش‌ها: مطالعه در زمستان 1389 انجام شد. نمونه‌برداري به تعداد 30 نمونه (15 نمونه آب و 15 نمونه رسوب) از 5 ایستگاه (4 ايستگاه در خليج گرگان و 1 ايستگاه در مصب رودخانه گرگانرود) صورت گرفت. در نمونه‌هاي جمع‌آوري شده غلظت فلزات سنگین روي، سرب، کروم و کادميوم اندازه‌گيري شد. براي اندازه‌گيري غلظت فلزات از دستگاه طيف‌سنجي جذب اتمي استفاده شد. یافته‌ها: یافته‌های تحقيق تفاوت معني‌داري را در غلظت فلزات سنگين موجود در آب و رسوب ايستگاه‌هاي مختلف نشان داد. بیشترین میزان غلظت فلزات سنگين در آب و رسوب مصب رودخانه گرگانرود و مصب رودخانه قره‌سو (ایستگاه‌های 1 و 2) به دست آمد. کمترین غلظت نیز در آب و رسوب ساحل گلوگاه و کانال خزینی (ایستگاه‌های 4 و 5) مشاهده شد. در بين فلزات مطالعه شده، فلز سرب بيشترين مقدار را در آب و فلز روي بيشترين مقدار را در رسوب داشت. همچنین مقايسه نتايج با مقدار حد مجاز آن‌ها در آب نشان داد که میزان کروم، کادمیوم و سرب در زمان انجام تحقيق بیشتر از حد مجاز Word health organization (WHO) است. نتایج غلظت فلزات در رسوب نیز نشان داد که غلظت فلزات پایین‌تر از مقدار آن در رسوب جهانی است. نتیجه‌گیری: با توجه به گسترش روزافزون جمعیت و افزایش میزان آلودگی‌ها ممکن است تغییراتی در غلظت فلزات سنگین مشاهده شود. بنابراین اندازه‌گیری و پایش غلظت فلزات سنگین باید به طور مستمر صورت گیرد.

Title: بررسی وضعيت حمايت خانواده در بيماران مبتلا به ديابت نوع دو و عوامل مرتبط با آن

http://hsr.mui.ac.ir/index.php/jhsr/article/view/290

مقدمه: حمایت اجتماعی به عنوان یک عامل بسیار مؤثر برای کنار آمدن موفق و آسان با شرایط و موقعیت‌های تنش‌زا شناخته شده است. با توجه به اهمیت مراقبت‌های درمانی در منزل از بیماران دیابت نوع 2، عملکرد خانواده در کیفیت رفتارهای سازگاری بیماران با مشکلات و پیامدها نقش اساسی دارد. بنابراین هدف اصلی پژوهش، تعیین وضعیت حمایت خانواده و برخی عوامل مرتبط با آن بود.  روش‌ها: مطالعه حاضر از نوع مقطعی و شیوه نمونه‌گیری در این پژوهش به صورت تصادفی ساده و مستمر بود. به این ترتیب که پژوهشگر زمان مراجعه را در روزهای مختلف هفته به صورت تصادفی انتخاب کرده و در مرحله بعد در زمان حضور در مرکز، نمونه‌گیری به روش آسان انجام شد. نمونه شامل 113 بیمار مراجعه کننده به مرکز تحقیقات صدیقه طاهره بود. برای تکمیل پرسش‌نامه در روزهای از پیش تعیین شده (به صورت تصادفی)، یک مصاحبه فردی با هر کدام از بیماران مراجعه کننده به مرکز انجام شد. برای ارزیابی داده‌ها از روش‌های همبستگی Pearson و ANOVA با اندازه‌های تکراری استفاده شد. یافته‌ها: تعداد 106 نفر (81 زن و 25 مرد) از مجموع 113 بیمار وارد تحلیل شدند. نتایج آزمون ANOVA، تفاوت معنی‌داری بین حمایت خانواده و سطح وضعیت اقتصادی نشان داد (001/0 > P، 24/3 = F). آزمون تحلیل واریانس با اندازه‌های تکراری، بین جنبه‌های حمایت خانواده درک شده از رفتارهای خودمراقبتی بیماران تفاوت معنی‌داری نشان داد (001/0 > P، 31/39 = F). نتایج تحلیل نشان داد که کمترین و بیشترین نمره گزارش شده از حمایت درک شده، به ترتیب مربوط به رفتار مصرف دارو و حمایت کلی بوده است. نتیجه‌گیری: حمایت خانواده درک شده بیماران نسبت به رفتار مصرف دارو و تبعیت از رژیم غذایی ضعیف بوده است. به دلیل اهمیت این دو جنبه در برنامه درمانی بیماران، ضروری است که توانمندسازی خانواده در برنامه‌های آموزشی مرتبط مورد توجه قرار گیرد.

Title: بررسی آلودگی صوتی ناشی از فرکانس‌های پایین صدا در یک کانال

http://hsr.mui.ac.ir/index.php/jhsr/article/view/350

مقدمه: در قرن حاضر صدا از مهم‌ترین معضلات زیست‌محیطی جوامع صنعتی و حتی کشورهای در حال توسعه می‌باشد. بر اساس گزارش سازمان بهداشت جهانی، در کشورهای اتحادیه اروپا حدود 40 درصد از جمعیت در معرض مواجهه با صدای ترافیک جاده‌ای با تراز فشار صوت معادل بیش از 55 دسی‌بل در طول روز می‌باشند. روش‌ها: این مطالعه از نوع تجربی است و از نرم‌افزار محاسباتی مطلب (MATLAB) استفاده شد که از قویترین نرم‌افزارهای پردازش و تولید سیگنال می‌باشد. ابتدا سیگنال در فركانس‌های 50 تا 500 هرتز و 1000 تا 6000 هرتز تولید شد. سپس از طریق بلندگو در طول کانالی به ابعاد دهانه 20 × 20 و طول 5/1 متر پخش شد. این سیگنال‌ها به ترتیب و پس از انتقال به کانال با نرخ نمونه‌برداری 8000 هرتز و زمان 10 ثانیه نمونه‌برداری شد. یافته‌ها: نتایج نشان داد که دامنه صدای کم فرکانس در فرکانس 500 به مقدار 05/0 و در فرکانس 250 هرتز به 15/0 افزایش یافت. در فرکانس‌های 1000، 4000 و 6000 به ترتیب 02/0، 01/0 و 004/0 کاهش دامنه مشاهده شد. با نگاه اول به نمودارها می‌توان تشخیص داد که با افزایش فرکانس، دامنه کاهش می‌یابد. این در حالی است که دامنه فرکانسی صدای کم (Low frequency noise و یا LFN) افزایش می‌یابد. نتیجه‌گیری: با نگاه کلی به نمودارها یا اطلاعات به دست آمده می‌توان استنباط نمود که بالا بودن قدرت صدای کم فرکانس در سیستم تهویه و توزیع آن از طریق کانال مسبب عوارض مشخص و جبران‌ناپذیر است. به هر حال می‌توان به صراحت موضوع تقویت صدای فرکانس پایین در کانال و سیستم تهویه را تأیید نمود. فرکانس‌های پایین صدا در کانال توزیع می‌شوند و البته با افزایش طول کانال به حداکثر دامنه خود خواهند رسید.

Title: تأثیر برنامه آموزش بهداشت بر اساس الگوی پرسید (Precede) بر آگاهی، نگرش و رفتار تغذیه‌ای مرتبط با کم‌خونی فقر آهن در دانش‌آموزان دختر مقطع دوم راهنمایی شهر اصفهان

http://hsr.mui.ac.ir/index.php/jhsr/article/view/364

مقدمه: کم‌خونی فقر آهن شایع‌ترین علت آنمی است که حدود 30 درصد جعیت جهان درگیر آن هستند. دختران نوجوان بالاترین میزان شیوع را دارند و چون عوارض آن بر بازده کار، نیروی تولید و در نهایت بر روند توسعه مؤثر است، پیش‌گیری از آن حایز اهمیت است. بنابراین این مطالعه با هدف تعیین تأثیر به کارگیری الگوی پرسید (Precede) به عنوان یک چارچوب نظری برای کنترل کم‌خونی فقر آهن در میان دانش‌آموزان دختر مقطع راهنمایی انجام شد. روش‌ها: پژوهش نیمه تجربی حاضر بر روی 130 نفر از دانش‌آموزان (65 نفر مورد و 65 نفر شاهد) که به طور تصادفی انتخاب شده بودند در سال 89-1388 انجام شد. مداخله در سه جلسه 60 دقیقه‌ای برگزار گردید و بلافاصله و سه ماه بعد از مداخله آموزشی توزیع و تکمیل پرسش‌نامه‌ها صورت گرفت. ابزار جمع‌آوری اطلاعات پرسش‌نامه‌ای محقق ساخته بر اساس اجزای مدل پرسید بود. به منظور دستیابی به نتایج از نرم‌افزارSPSS  نسخه 18 و آزمون‌های آماری Independent t، Paired t، ANOVA with repeated measures و 2c استفاده گردید. یافته‌ها: پس از مداخله آموزشی در گروه مورد نسبت به گروه شاهد میانگین نمره آگاهی، میانگین نمره نگرش، تشکیل کلاس‌های آموزشی، شرکت در کلاس‌های آموزشی و استفاده از منابع آموزشی (عوامل قادر کننده)، تشویق خانواده و معلمان (عوامل تقویت کننده) و رفتارهای پیش‌گیری کننده از کم‌خونی فقر آهن افزایش معنی‌داری یافتند (001/0 > P). نتیجه‌گیری: مطالعه حاضر نشان دهنده تأثیر مثبت مداخله آموزشی بر اساس الگوی پرسید و اجزای اصلی آن (عوامل مستعد کننده، عوامل قادر کننده و عوامل تقویت کننده) در افزایش رفتارهای پیشگیری کننده از کم‌خونی فقر آهن در جامعه مورد مطالعه بود.

Title: ارزیابی سیستم‌های اسمز معکوس در بهبود کیفیت آب مورد استفاده در مایع همودیالیز بیمارستان‌های شهر اصفهان

http://hsr.mui.ac.ir/index.php/jhsr/article/view/546

مقدمه: کیفیت شیمیایی آب مورد استفاده در تهیه مایع دیالیز برای درمان بیماران همودیالیزی فاکتور بسیار مهمی است. از این رو آب مورد استفاده در تهیه مایع دیالیز را باید قبل از استفاده با روش‌های اختصاصی تصفیه نمود. هدف این مطالعه تعیین کارایی سیستم‌های اسمز معکوس در بهبود کیفیت آب مورد استفاده در تهیه مایع دیالیز است. روش‌ها: در این مطالعه توصیفی 40 نمونه آب از ورودی و خروجی از اسمز معکوس 5 بیمارستان شهر اصفهان در طی ماه‌های فروردین تا مرداد سال 1390 جمع‌آوری شد. سپس از نظر پارامترهای فیزیکوشیمیایی کلر باقی‌مانده، کلرامین، pH، سختی، آلومینیم، فلوئور، مس، منگنز، کادمیوم، کروم، سرب و نیکل با استفاده از روش‌های استاندارد آزمایش‌ آب بررسی گردید. یافته‌ها: سیستم اسمز معکوس به طور معنی‌داری میزان کلر باقی‌مانده، کلرامین، سختی، سدیم، پتاسیم، فلوراید، نیترات، سولفات و منگنز آب خروجی از اسمز معکوس را کاهش داد، ولی تأثیر معنی‌داری بر کاهش میزان مس، روی، کادمیوم، کروم، سرب و آلومینیم نداشت. همچنین میزان نیترات، سختی، سرب، کادمیوم، کروم و آلومینیم در آب خروجی از اسمز معکوس از استاندارد آب همودیالیز بالاتر بود. نتیجه‌گیری: سیستم اسمز معکوس می‌تواند کیفیت آب مورد استفاده برای همودیالیز را بهبود دهد، اما نمی‌تواند سطح برخی از آلاینده‌ها را تا مقادیر توصیه شده برای مایع همودیالیز کاهش دهد. از این رو استفاده از این آب می‌تواند برای استفاده همودیالیز ایمن نباشد. بنابراین استفاده از روش‌های ترکیبی برای کنترل آلاینده‌های شیمیایی و اطمینان از همودیالیز با کیفیت خوب ضروری است.

Title: بررسی نیازهای آموزشی داوطلبین ازدواج شهر قم نسبت به تالاسمی و عوامل مرتبط با آن: یک مطالعه مقطعی

http://hsr.mui.ac.ir/index.php/jhsr/article/view/553

مقدمه: تالاسمی شایع‌ترین بیماری ژنتیک در انسان است و به عنوان یک مشکل بهداشتی در سراسر جهان رو به گسترش است. به علت اهمیت این بیماری در کشور ما برنامه‌هاي پيش‌گيري از تالاسمي دارای اهميت قابل توجهی می‌باشد. این مطالعه با هدف، بررسی نیازهای آموزشی داوطلبین ازدواج شهر قم نسبت به تالاسمی و عوامل مرتبط با آن انجام شد. روش‌ها: مطالعه مقطعی حاضر بر روی 420 داوطلب ازدواج مراجعه کننده به مرکز مشاوره امیرالمؤمنین قم صورت پذیرفت. روش نمونه‌گیری از نوع مبتنی بر هدف و در دسترس بود. جمع‌آوری اطلاعات از طریق پرسش‌نامه‌ای مشتمل بر 36 سؤال صورت پذیرفت. داده‌ها پس از جمع‌آوري با نرم‌افزار SPSS نسخه 16 مورد تجزيه و تحليل قرار گرفت. در این مطالعه سطح معنی‌داری 05/0 > P در نظر گرفته شد. یافته‌ها: میانگین سن ازدواج در آقایان 35/25 و در خانم‌ها 81/20 بود. رادیو و تلویزیون به عنوان مهم‌ترین منبع کسب اطلاعات پیرامون تالاسمی ذکر شد. در مجموع، 7/20 درصد آگاهی خوب، 9/42 درصد آگاهی متوسط و 4/36 درصد آگاهی ضعیفی درباره تالاسمی داشتند. همچنین از نظر سطح نگرش، 15 درصد نگرش خوب، 61 درصد نگرش متوسط و 24 درصد نگرش ضعیف نسبت به پیش‌گیری و ضرورت مشاوره ازدواج داشتند. تفاوت آماری معنی‌داری از نظر آگاهی و نگرش در مردان و زنان مورد مطالعه مشاهده نشد (05/0 < P). نتیجه‌گیری: با توجه به این که آگاهی و نگرش داوطلبین ازدواج در مورد تالاسمی، از میزان مناسبی برخوردار نبود، طراحی مداخلات آموزشی چند جانبه در این زمینه ضروری می‌نماید.

Title: بررسي وضعيت سلامت رواني و عوامل مؤثر بر آن در ميان دانشجويان دانشگاه ايلام

http://hsr.mui.ac.ir/index.php/jhsr/article/view/555

مقدمه: دانشجويان به ويژه آن‌هايي كه در اواخر دوران نوجواني و اوايل دوره جواني هستند و تازه وارد دانشگاه شده‌اند، به دلیل ماهيت انتقالي زندگيشان در معرض فشار و استرس بوده و عوامل استرس‌زاي بي‌شماري را تجربه مي‌كنند كه سلامت رواني آن‌ها را در معرض تهديد قرار مي‌دهد. به همین منظور تحقیق حاضر با هدف، بررسی وضعیت سلامت روانی و عوامل مؤثر بر آن در میان دانشجویان دانشگاه ایلام انجام گرفت. روش‌ها: شركت كنندگان این مطالعه، دانشجويان كارداني، كارشناسي و كارشناسي ارشد ورودي سال 1389 دانشگاه ایلام بودند که تعداد آن‌ها 661 نفر بود و 288 نفر از آن‌ها را پسر و 373 را دختر تشکیل می‌دادند. ابزار مورد استفاده در اين پژوهش، پرسش‌نامه سلامت روان بود كه توسط كارشناسان دفتر مركزي مشاوره وزارت علوم طراحي شده است. متغيرهاي دموگرافيک مؤثر بر سلامت روان نیز در اين پرسش‌نامه گنجانده شده است. در اين پژوهش نقطه برش 100 جهت شناسايي افرادي كه نيازمند اقدامات روان‌شناختي و روان درماني بودند، تعیین شد. آزمون‌هاي آماری Friedman، ضرايب همبستگي رگرسيون، ANOVA و آزمون t جهت آمار تحلیلی و نرم‌افزار SPSS نسخه 16 جهت تجزیه و تحلیل نهایی داده‌ها مورد استفاده قرار گرفت. یافته‌ها: 17 درصد از دانشجويان ورودي سال 1389 در معرض خطر بودند. نتایج حاکی از رابطه معني‌دار بين رشته تحصيلي و علاقه به رشته تحصيلي با سلامت روان بود. همچنين بيشترين و كمترين ميانگين‌هاي رتبه‌اي به ترتيب اضطراب اجتماعي و اضطراب بودند. نتيجه ضرايب رگرسيون نشان داد كه همه عوامل (افسردگي، اضطراب، وسواس، اضطراب اجتماعي و اختلال خواب) رابطه معني‌داري با نمره كل آسيب‌شناسي دانشجويان دارند (01/0 > P). یافته‌ها نشان داد كه تمايل به خودكشي با نمره كل آسيب‌شناسي فرد رابطه معني‌ دار دارد. تمايل به خودكشي رابطه منفي معني‌داری با حمايت اجتماعي دارد. گرايش به خودكشي نیز با خودكارامدي و كمال‌طلبي رابطه دارد. حمايت اجتماعي با خودكارامدي رابطه معني‌دار دارد. همچنين، خودكارامدي رابطه معني‌داري با كمال‌طلبي دارد. همه همبستگي‌ها در سطح 01/0 و 05/0 معني‌دار بودند. نتیجه‌گیری: لزوم توجه به مشكلات در حال افزايش سلامت روان در بين دانشجويان و توجه جدي به آن‌ها باید به صورت جدی مورد توجه قرار گیرد.

Title: بررسی موانع درک شده و رفتار پرستاران بیمارستان‌های آموزشی دانشگاه علوم پزشکی لرستان در مورد گزارش خطاهای دارویی

http://hsr.mui.ac.ir/index.php/jhsr/article/view/578

مقدمه: خطاهای دارویی از شایع‌ترین خطاهای پزشکی بوده و یکی از چالش‌های مهم تهدید کننده نظام سلامت محسوب می‌شود. این مطالعه با هدف، موانع درک شده گزارش خطاهای دارویی و رفتار پرستاران در مورد خطاهای دارویی در بیمارستان‌های آموزشی لرستان در سال 1390 انجام شد. روش‌ها: تعداد 403 پرستار به روش نمونه‌گیری آسان از بیمارستان‌های آموزشی دانشگاه علوم پزشکی لرستان در شهر خرم‌آباد انتخاب و پرسش‌نامه استاندارد اصلاح شده Gladstone در ایستگاه پرستاری توزیع و در انتهای شیفت جمع‌آوری گردید. داده‌ها با استفاده از نرم‌افزار آماری SPSS نسخه 16 و آزمون‌های آماری 2c، Independent t و ANOVA مورد تجزیه تحلیل قرار گرفت. یافته‌ها: 73 درصد (294 نفر) پرستاران از این که چه مواردی به عنوان خطای دارویی محسوب می‌شود، مطمئن نبودند. 6/81 درصد (329 نفر) پرستاران گزارش دادند که ترس از واکنش مدیر پرستاری مانع اصلی گزارش‌دهی می‌باشد و 2/38 درصد (154 نفر) پرستاران خطای دارویی مرتکب شده را از ترس از دست دادن شغل گزارش نکرده بودند. در رتبه‌بندی علل خطای دارویی، پرستاران بیشترین اهمیت را به ناخوانا بودن دستور پزشک و کمترین رتبه را به آسیب‌دیدگی برچسب دارویی دادند. بین رتبه‌بندی علل خطا از دیدگاه پرستاران و متغیرهای جنس و سابقه کاری، ارتباط معنی‌داری مشاهده شد (05/0 = P). نتیجه‌گیری: برگزاری دوره‌های آموزشی جهت افزایش توان علمی و انگیزش پرستاران برای توجه به اصل سوددهی به بیمار و ایجاد سیستم مناسب گزارش‌دهی خطای دارویی که در آن پرستار فارغ از دغدغه‌های فکری و شغلی باشد، ضروری است.

Title: بررسی پیش‌گویی کنندگی رفتارهای خودمراقبتی بر کنترل قند خون بیماران مبتلا به دیابت نوع 2

http://hsr.mui.ac.ir/index.php/jhsr/article/view/579

مقدمه: بیماری دیابت درمان قطعی ندارد. مهم‌ترین استراتژی درمانی به کار گرفته شده برای کنترل آن، تبعیت از رفتارهای خودمراقبتی شامل رعایت رژیم غذایی ویژه، انجام فعالیت بدنی، پایش قند خون، مصرف روزانه داروهای تجویز شده و مراقبت از پا می‌باشد. هدف از مطالعه حاضر، شناسایی تفکیکی میزان تبعیت از ابعاد مختلف رفتارهای خودمراقبتی و ارزیابی قدرت پیش‌بینی کنندگی هر یک از این ابعاد بر کنترل قند خون در بیماران مبتلا به دیابت نوع 2 تحت پوشش کلینیک دیابت دانشگاه علوم پزشکی قم بود. روش‌ها: مطالعه توصیفی- تحلیلی حاضر به صورت یک مطالعه مقطعی بر روی 275 نفر از بیماران مبتلا به دیابت نوع 2 که به صورت تصادفی انتخاب شده بودند و شرایط ورود به مطالعه را داشتند، انجام شد. برای جمع‌آوری داده‌ها از «مقیاس خلاصه فعالیت‌های خودمراقبتی»، پرسش‌نامه ارزیابی سلامت شناختی، پرسش‌نامه ارزیابی افسردگی Beck و نیز آزمون (HbA1c) Glycosylated Hemoglobin استفاده گردید. آزمون‌های آماری ضریب همبستگی دو متغیره و رگرسیون خطی چند متغیره برای تحلیل داده‌ها و نرم‌افزار SPSS نسخه 17 برای تجزیه نهایی آن‌ها مورد استفاده قرار گرفت. یافته‌ها: مدل نهایی رگرسیون شامل متغیرهای پیش‌بین سطح تحصیلات، رفتارهای بعد مصرف دارو، تغذیه، فعالیت بدنی و پایش قند خون بوده و مدل پیشنهادی توانست 66 درصد واریانس متغیر HbA1c را به عنوان شاخص کنترل قند خون پوشش دهد (001/0 > P). نتیجه‌گیری: در این مطاله ابعاد مختلف رفتارهای خودمراقبتی بیماران مبتلا به دیابت نوع 2 تأثیر متفاوتی بر کنترل قند خون داشته و در بین آن‌ها رفتارهای خودمراقبتی بعد مصرف دارو و تغذیه بیشترین تأثیر را بر آن داشتند.

Title: ارتقای فعالیت بدنی بیماران مبتلا به دیابت نوع 2: یک مداخله تئوری محور

http://hsr.mui.ac.ir/index.php/jhsr/article/view/585

مقدمه: فعالیت بدنی منظم خطر مرگ و میر در افراد مبتلا به دیابت را کاهش می‌دهد و از بروز عوامل ایجاد کننده بیماری‌های قلبی و عروقی جلوگیری می‌نماید. طراحی برنامه‌های تغییر رفتار در این افراد به منظور افزایش مهارت‌های رفتاری و ارتقای رفتارهای خودمراقبتی از اهمیت بسیار بالایی برخوردار می‌باشد. بنابراین پژوهش حاضر با هدف، بررسی تأثیر 8 هفته مداخله آموزشی طراحی شده بر اساس ترنس تئورتیکل مدل (Trans-theoretical model یا TTM) در ارتقای فعالیت بدنی بیماران مبتلا به دیابت نوع 2 شهر همدان انجام گرفت. روش‌ها: در یک مطالعه مداخله‌ای، 50 بیمار مبتلا به دیابت نوع 2 با میانگین سنی 64/6 ± 07/50 سال که از فعالیت بدنی منظم برخوردار نبودند و در مراحل پیش عملیاتی مدل مراحل تغییر قرار داشتند، شرکت کردند و به روش تصادفی به دو گروه مداخله (2 نفر) و شاهد (25 نفر) تقسیم شدند و پرسش‌نامه سنجش سازه‌های TTM را تکمیل نمودند. سپس برنامه آموزشی 8 هفته‌ای انجام فعالیت بدنی منظم برای گروه مداخله اجرا گردید؛ در حالی که گروه شاهد فقط آموزش‌های روزانه مرکز تحقیقات دیابت را دریافت می‌نمودند. بعد از یک ماه پیگیری، بیماران دوباره پرسش‌نامه‌های مربوط به پژوهش را تکمیل کردند. اطلاعات جمع‌آوری شده با استفاده از نرم‌افزار آماری SPSS نسخه 16 مورد تجزیه و تحلیل قرار گرفت. یافته‌ها: نتایج مطالعه نشان داد که بین گروه مداخله و شاهد قبل از انجام مداخله آموزشی هیچ گونه اختلاف معنی‌داری در انجام فعالیت بدنی منظم و سازه‌‌های TTM وجود نداشت. بعد از مداخله آموزشی، مراحل تغییر انجام فعالیت بدنی در گروه مداخله به طور معنی‌داری در مقایسه با گروه شاهد افزایش یافت (05/0 > P). همچنین نتایج نشان دهنده افزایش معنی‌دار در میانگین سازه‌های فرایندهای شناختی، فرایندهای رفتاری، تغییر و خودکارامدی درگروه مداخله در مقایسه با گروه شاهد بعد از انجام مداخله آموزشی بود (05/0 > P)، اما افزایش میانگین سازه توازن و تعادل در تصمیم‌گیری در گروه مداخله نسبت به گروه شاهد معنی‌دار نبود (05/0 < P). تفاوت معنی‌داری بین گروه مداخله و شاهد در به کارگیری سطوح فرایندهای تغییر شناختی و رفتاری بعد از مداخله آموزشی مشاهده گردید (05/0 > P)، اما این تفاوت در سطح خودارزیابی مجدد معنی‌دار نبود (05/0 < P). نتیجه‌گیری: نتايج پژوهش نشان داد كه آموزش بیماران مبتلا به دیابت در زمینه انجام فعاليت بدنی بر اساس TTM در پیشرفت مرحله تغییر و اتخاذ رفتار فعالیت بدنی منظم بيماران مؤثر است. بنابراین طراحی و اجرای برنامه‌های آموزشی برای ارتقای فعاليت‌هاي بدنی بر اساس تئوری‌های تغییر رفتار به منظور بهبود کنترل و مدیریت دیابت نوع 2 توصيه مي‌شود.

Title: سبک زندگی مرتبط با اینترنت در دانشجویان کاربر اینترنت دانشگاه‌های شهر گرگان

http://hsr.mui.ac.ir/index.php/jhsr/article/view/619

مقدمه: اینترنت در سالیان اخیر گسترش چشمگیری در ایران داشته است. دانشجویان از کاربران اصلی اینترنت محسوب می‌شوند. در این شرایط این پرسش به وجود می‌آید که اینترنت چه تأثیری بر سبک زندگی این گروه از کاربران دارد و باعث کدام تغییرات رفتاری در این حیطه می‌شود. این مطالعه با هدف بررسی سبک زندگی و ارتباط آن با استفاده از اینترنت در دانشجویان کاربر آن انجام شد. روش‌ها: این مطالعه در سال 1388 به صورت مقطعی در بین 600 دانشجوی دانشگاه‌های شهر گرگان انجام شد. نمونه‌ها با روش نمونه‌گیری مرحله‌ای چند طبقه‌ای انتخاب شدند. اطلاعات به وسیله پرسش‌نامه‌ای جمع‌آوری گردید که با تعیین پایایی و روایی تنظیم شده بود. توصیف داده‌ها با نرم‌افزار آماری 5/11SPPS انجام شد. یافته‌ها: اکثریت شرکت‌کنندگان رفتار تغذیه‌ای مناسبی داشتند. بیش از 50 درصد اعلام کردند که اینترنت تغییری در وضعیت تغذیه آن‌ها نمی‌دهد. 4/44 درصد بیان کردند که در یک ماه گذشته اصلاً ورزش نکردند. 4/68 درصد گفتند که اینترنت تغییری در وضعیت فعالیت بدنی آن‌ها ندارد. 9/18 درصد احساس خستگی و 8/32 درصد احساس آرامش و رضایتمندی بعد از کار با اینترنت را گزارش نمودند. تقریباً 90 درصد افراد به این سؤال که اینترنت چه تأثیری بر وضعیت مصرف سیگار، مواد مخدر و رابطه جنسی غیر ایمن در آن‌ها داشته است، پاسخ ندادند. نتیجه‌گیری: به نظر نمی‌رسد اینترنت بر سبک زندگی دانشجویان مورد بررسی مؤثر بوده باشد. با توجه به افزایش روزافزون استفاده از اینترنت در این گروه از کاربران ایرانی، نیاز به مطالعه‌های بیشتر در زمینه تأثیر اینترنت بر سبک زندگی احساس می‌شود.

Title: ارزیابی اثربخشی برنامه جرأت‌ورزی بر میزان استرس، اضطراب و افسردگی دانش‌آموزان دبیرستانی

http://hsr.mui.ac.ir/index.php/jhsr/article/view/369

مقدمه: از ویژگی‌های دوره نوجوانی می‌توان به شکل‌گیری و رشد عاطفی، اجتماعی و شناختی اشاره کرد. در این دوره، نوجوان در وضعیتی بین دو مرحله کودکی و بزرگسالی و زیر فشار و انتظارات این وضعیت پیچیده و مبهم قرار می‌گیرد. حالت هیجانی در دوره بلوغ غنی‌تر می‌گردد و گاهی به صورت حساسیت و هیجان شدید در می‌آید؛ به طوری که زودرنجی، افسردگی، اضطراب و پرخاشگری در بیشتر نوجوانان به چشم می‌خورد. جرأت‌ورزی يک شيوه مداخله ساخت يافته است که از آن براي بهبود اثربخشي روابط اجتماعي استفاده مي‌شود. این مطالعه با هدف تعیین تأثیر برنامه جرأت‌ورزی بر استرس، اضطراب و افسردگی دانش‌آموزان دبیرستانی انجام شد. روش‌ها: اين مطالعه يک مطالعه مداخله‌اي نیمه تجربی است که در سال تحصیلی 90-1389 در دبیرستان‌های شهر اصفهان صورت گرفت. در این پژوهش 126 دانش‌آموز پایه دوم متوسطه در دو گروه آزمون (63 نفر) و گروه شاهد (63 نفر) انتخاب شدند. ابزار جمع‌آوری داده‌ها شامل پرسش‌نامه اطلاعات دموگرافيک، پرسش‌نامه‌هاي جرأت‌ورزی Gambrill و Richey و 21DASS بود. برنامه جرأت‌ورزی طی 7 جلسه به گروه آزمون آموزش داده شد و پس از 8 هفته در دو گروه پس آزمون گرفته شد. سپس تجزيه و تحليل اطلاعات با استفاده ازآزمون‌هاى آمارى Independent t، آزمون آنالیز واریانس با تکرار مشاهدات، 2c و Mann-Whitney صورت گرفت. یافته‌ها: با توجه به یافته‌های به دست آمده، آزمون آماری 2c و Mann-Whitney اختلاف معنی‌داری را از نظر متغیرهای دموگرافیکی بین دو گروه آزمون و شاهد نشان نداد. آزمون آنالیز واریانس با تکرار مشاهدات نشان داد که بین میانگین نمره جرأت‌ورزی قبل، بلافاصله و 3 ماه پس از مداخله در گروه شاهد اختلاف معنی‌داری وجود نداشت. همین آزمون میانگین نمره جرأت‌ورزی در گروه مورد را در زمان‌های مختلف دارای اختلاف معنی‌دار نشان داد (002/0 P =). همچنین آزمون Independent t نشان داد که میانگین نمره اضطراب و استرس در زمینه جرأت‌ورزی دانش‌آموزان در دو گروه قبل از مداخله اختلاف معنی‌داری نداشتند، اما بلافاصله و 2 ماه بعد از آموزش میانگین نمره اضطراب در گروه مورد به طور معنی‌داری بیشتر از گروه شاهد بود. در مورد نمره افسردگی در زمینه جرأت‌ورزی در دانش‌آموزان، آزمون Independent t قبل از آموزش در دو گروه اختلاف معنی‌داری را نشان نداد. بعد از آموزش با وجود کاهش نمره افسردگی در گروه مورد، این اختلاف معنی‌دار نبود (09/0 P =). نتیجه‌گیری: نتایج حاصل نشان داد که اجرای برنامه‌های جرأت‌آموزی در دانش‌آموزان سنین دبیرستانی منجر به کاهش میزان اضطراب، استرس و افسردگی آنان می‌شود. با توجه به این که سنین دبیرستان یکی از حساس‌ترین سنین دوران زندگی هر فردی است و با توجه به ماهیت این گونه برنامه‌ها که در عین ارزان و ایمن بودن، کارا و اثربخش نیز می‌باشند، اجرای آن را در جهت کنترل استرس، اضطراب و افسردگی نوجوانان دبیرستانی توصیه می‌گردد.

Title: شهروندی در مدارس: یک مطالعه توصیفی مبتنی بر نظریه عمل منطقی

http://hsr.mui.ac.ir/index.php/jhsr/article/view/663

مقدمه: امروزه آموزش جامع شهروندی به عنوان راهبردی مهم جهت توانمندسازی جوانان برای بر عهده گرفتن مسؤولیت‌ها و ایفای نقش‌های فردی و اجتماعی و همچنین صیانت آنان در مقابل آسیب‌های روانی- اجتماعی مورد توجه و تأکید صاحب‌نظران توسعه اجتماعی قرار دارد. هدف از این مطالعه، سنجش میزان دانش، نگرش، هنجار انتزاعی و قصد رفتاری دانش‌آموزان در خصوص مهارت‌ها و ویژگی‌های شهروندی بود. روش‌ها: از بین دبیرستان‌های دختر ناحیه سه شهر شیراز که حداقل سه رشته تحصیلی (علوم ریاضی، علوم تجربی و علوم انسانی) در آن‌ها دایر بود (تعداد =١٢)، سه مدرسه به طور تصادفی انتخاب شدند. در هر مدرسه یک کلاس از هر رشته به روش تصادفی ساده انتخاب و در مجموع 228 دانش‌آموز در مطالعه شرکت داده شدند. یک پرسش‌نامه 62 گویه‌ای، مشتمل بر 15 سؤال چند گزینه‌ای برای آزمون دانش و 34 سؤال با مقیاس لیکرت برای اندازه‌گیری سازه‌های نظریه به عنوان داده‌های پژوهش استفاده گردید. روایی محتوایی با استفاده از نظرات ٧ نفر از متخصصان در رشته‌های آموزش بهداشت، جامعه‌شناسی و علوم اجتماعی، بررسی و پس از ویرایش مورد تأیید آنان قرار گرفت. پایایی ابزار نیز از طریق اجرای مطالعه راهنما (پایلوت) و با استفاده از شیوه بازآزمایی اندازه‌گیری شد. ضرایب همبستگی سازه‌‌های مطالعه شامل دانش (82/0 = r)، نگرش (97/0 = r)، هنجار انتزاعی (94/0 = r) و قصد رفتاری (97/0 = r) تأیید گردید. یافته‌ها: یافته‌های مطالعه نشان داد که سطح دانش (٩٨/١ ± ٨٤/٧) و نگرش (٤٤/٧ ± ٣٨/٧٥) دانش‌آموزان نسبتاً پایین بود. همچنین هنجار انتزاعی (٣٣/٤ ± ٢٤/٢٣) و قصد رفتاری (٠٣/٣ ± ٠٧/١٨) نسبتاً مطلوب، ولی کمتر از سطح ایده‌آل بود. تحلیل روابط بین متغیرهای مطالعه نشان داد که نگرش و دانش (139/0 = r، 037/0 = P)، نگرش و قصد رفتاری (199/0 = r، ٠003/0 = P)، هنجار انتزاعی و قصد رفتاری (143/0 = r،031/0 = P) و هنجار انتزاعی و نگرش (174/0 = r، ٠٠٨/٠ 008/0 = P) همبستگی قابل توجهی با یکدیگر داشتند. در مقابل بین متغیرهای جمعیت‌شناختی و سازه‌های اصلی مطالعه (دانش، نگرش، هنجار انتزاعی و قصد رفتاری) رابطه آماری معنی‌‌داری مشاهده نگردید. نتیجه‌گیری: پایین بودن نمرات نگرش، ضرورت برنامه‌ریزی آموزشی را با در نظر گرفتن ویژگی‌ها و شرایط مخاطبان دلالت می‌نماید. مطلوب بودن نسبی سطح قصد رفتاری و هنجار انتزاعی در این مطالعه پیش‌آگهی مطلوبی را برای اثربخشی مداخلات آموزشی مطرح می‌نماید. در نهایت طراحی و اجرای مداخلات آموزشی مبتنی بر نظریه عمل استدلالی برای آموزش شهروندی در مدارس پیشنهاد می‌گردد.

Title: مقایسه تنش قلبی کارگران با وزن نرمال و اضافه وزن در شرایط جوی گرم و مرطوب جنوب ایران

http://hsr.mui.ac.ir/index.php/jhsr/article/view/349

مقدمه: در شرایط جوی گرم محیط کار، اضافه وزن و چاقی به عنوان یک عامل خطر در بروز تنش قلبی کارگران مطرح است. هدف از این مطالعه، مقایسه تنش قلبی در دو گروه با وزن نرمال و اضافه وزن در شرایط گرم و مرطوب جنوب ایران بود. روش‌ها: مطالعه مقطعی حاضر بر روی 71 نفر از کارگران ساختمانی، نصب تجهیزات فنی، جوشکار و ناظر در منطقه پارس جنوبی ایران در تابستان سال 1389 انجام گرفت. ضربان قلب در دوره استراحت (Resting heart rate یا RHR) برابر با 9/1 ± 6/22 و در زمان انجام کار برابر با 0/2 ± 3/33 شاخص دمای تر گویسان (Wet bulb globe temperature یا WBGT) اندازه‌گیری شد. شدت تنش قلبی بر حسب ضربان قلب کار (Working heart rate یا WHR)، هزینه نسبی قلبی (Relative cardiac cost یا RCC)، هزینه خالص قلبی (Net cardiac cost یا NCC) و نرخ کاهش ضربان قلب در 35 نفر با وزن نرمال (شاخص توده بدنی کمتر از 25) و 36 نفر با اضافه وزن و چاق (شاخص توده بدنی بیشتر از 25) با کاربرد آمار توصیفی و آزمون t مورد تحلیل قرار گرفت. یافته‌ها: میانگین شاخص توده بدنی (Body mass index یا BMI)، 4 ± 25 کیلوگرم بر مترمربع بود. BMI در 42 درصد کارگران بالاتر از 25 بود. میانگین RHR در افراد دارای وزن نرمال و اضافه وزن به ترتیب 9/11 ± 70 و 6/9± 75 و میانگین WHR در دو گروه به ترتیب 3/20 ± 101 و 9/ 18± 112 به دست آمد (026/0 = P). درصد تجاوز پارامترهای NCC، RCC و WHR و شاخص برهه از حدود قابل قبول در گروه اضافه وزن نسبت به گروه وزن نرمال به طور قابل توجهی بالاتر بود. نتیجه‌گیری: بر اساس نتایج مطالعه، شدت تنش قلبی در کارگران دارای اضافه وزن در مقایسه با کارگران دارای وزن نرمال بالاتر بود. بنابراین برای کاهش تنش قلبی، از انتخاب افراد دارای اضافه وزن برای این مشاغل اجتناب شود و همچنین با انجام مداخله‌های ضروری برای کاهش وزن از قبیل آموزش‌های تغذیه‌ای و تشویق برای انجام منظم فعالیت بدنی، شدت تنش قلبی را کاهش داد.

Title: عادات و دریافت‌های غذایی در دانشجويان دختر دانشگاه علوم پزشکی اصفهان

http://hsr.mui.ac.ir/index.php/jhsr/article/view/899

مقدمه: از آن جایی که اصلاح دریافت‌های غذایی در گروه‌ها و جمعیت‌های مختلف به خصوص در دختران جوان اهمیت ویژه‌ای دارد، این مطالعه با هدف تعیین دریافت مواد مغذی، گروه‌های غذایی و نیز عادات غذايي در دانشجويان دختر دانشگاه علوم پزشکی اصفهان صورت گرفت. روش‌ها: مطالعه توصیفی- مقطعی حاضر بر روی 289 دانشجوي دختر سالم دانشگاه علوم پزشكي اصفهان انجام شد. برای انتخاب دانشجويان از نمونه‌گیری سیستماتیک خوشه‌ای تصادفي استفاده گردید. جهت برآورد دريافت‌های غذايي معمول از پرسش‌نامه اعتبارسنجی شده نیمه کمی بسامد خوراک استفاده شد. بررسی عادات غذایی با استفاده از پرسش‌نامه ارزیابی عادات غذایی انجام پذیرفت. یافته‌ها: میانگین سن شرکت کنندگان 7 ± 21 سال و ميانگين نمايه توده بدن (Body mass index یا BMI) 1/5 ± 9/25 کیلوگرم بر مترمربع بود. میانگین مصرف لبنیات کم‌چرب و پرچرب به ترتیب 11/0 ± 3/1 و 16/0 ± 3/1 سروینگ بود. دریافت روزانه غلات کامل تنها 2/0 ± 0/1 سروینگ بود؛ در حالی که غلات تصفیه شده 2/1 ± 0/6 سروینگ در روز مصرف می‌شد. مصرف میوه‌ها و سبزیجات 4/0 ± 2/2 و 0/1 ± 2/3 سروینگ در روز بود. فولات، آهن، کلسیم و فیبر غذایی کمتر از میزان مجاز توصیه شده روزانه (Recommended dietary allowance یا RDA) دریافت می‌شد. (به ترتیب 56، 90، 76، 70 درصد RDA). 45 درصد جمعیت مورد مطالعه 2 بار در هفته فست فود می‌خوردند. 35 درصد افراد بیشتر مواقع برای پخت غذا از روغن‌های سرخ کردنی استفاده می‌کردند و 52 درصد نیز وعده صبحانه را حذف کرده بودند. نتیجه‌گیری: نتایج این مطالعه نشان داد، عادات غذایی جمعیت مورد مطالعه نیاز به اصلاح دارد. توجه بیشتر به دریافت ریز مغذی‌ها از جمله فولات، آهن و دریافت بیشتر محصولات لبنی و فیبر غذایی در این گروه اهمیت ویژه‌ای دارد.

Title: کاربرد مدل آمیخته خطی نیرومند در بررسی رابطه نوبت کاری و تغییرات طولی کلسترول خون با کنترل تأثیر وزن هنگام استخدام

http://hsr.mui.ac.ir/index.php/jhsr/article/view/900

مقدمه: رابطه نوبت کاری و بیماری‌های مزمن مانند بیماری‌های قلبی- عروقی و مکانیزم‌های مرتبط در مطالعات متعددی مورد بررسی قرار گرفته است. هدف این مطالعه، بررسی رابطه نوبت کاری و تغییرات طولی کلسترول با کنترل اثر وزن هنگام استخدام بود. روش‌ها: این مطالعه یک هم‌گروهی گذشته‌نگر شامل 674 نفر از کارکنان مرد استخدام شده طی سال‌های 1370 تا 1387 در شرکت پلی‌اکریل اصفهان بود. رابطه نوبت کاری و کلسترول به تفکیک وضعیت وزن هنگام استخدام نسبت به متغیرهای مخدوشگر شامل سن، شاخص توده بدنی (Body mass index یا BMI)، میزان کلسترول در شروع استخدام، گلوکز، تری‌گلیسیرید، اوره، نوع کار، سطح تحصیلات و وضعیت تأهل کنترل شد. برای تجزیه و تحلیل داده‌ها از مدل آمیخته خطی با خطاهای بیضوی-چوله استفاده شد. برآورد پارامترها با استفاده از رهیافت بیزی (Bayesian approach) و در نرم‌افزار Winbugs صورت گرفت. بازه اطمینان بیزی به منظور آزمون ضرایب رگرسیونی به کار گرفته شد. یافته‌ها: با توجه به معیار اطلاع انحراف، مدل آمیخته خطی با خطای نرمال-چوله نسبت به دیگر مدل‌ها بهتر بود. بر اساس برآوردهای این مدل، رابطه بین نوبت کاری و تغییرات کلسترول با کنترل متغیرهای مخدوشگر در افرادی که در بدو استخدام دارای اضافه وزن نبودند، معنی‌دار بود (88/3، 67/0 = CI 95 درصد، 001/0 = P، 25/2 = b)، ولی در افرادی که اضافه وزن داشتند معنی‌دار نبود. نتیجه‌گیری: میانگین تغییرات کلسترول با افزایش سن در نوبت کارانی که هنگام استخدام اضافه وزن نداشتند به صورت معنی‌داری بیشتر از نوبت کارانی بود که هنگام استخدام دارای اضافه وزن بودند. همچنین مدل آمیخته خطی با خطاهای دم سنگین و چوله برازش بهتری نسبت به مدل آمیخته نرمال نشان داد.

Title: بررسی نگرش کارشناسان مدیریت جهاد کشاورزی شهرستان اصفهان پیرامون کاربرد فن‌آوری نانو در کشاورزی

http://hsr.mui.ac.ir/index.php/jhsr/article/view/901

مقدمه: فن‌آوري ‌نانو با کمک ابزارهاي جديد، توانايي دگرگون سازي صنايع غذايي و کشاورزي را دارد و مي‌تواند از اين ابزارها براي تشخيص رفتارهاي مولکولي بيماري‌ها، کشف سريع بيماري و افزايش توانايي گياهان براي جذب مواد غذايي استفاده کند. با توجه به کاربردهای وسیع این فن‌آوری در صنایع و بخش‌های مختلف، بخش كشاورزي از جمله مهم‌ترين عرصه‌‌هايي است كه با استفاده از دستاوردهاي فن‌آوري نانو، منافع زيادي را متوجه اين بخش خواهد نمود. با توجه به اهمیت کاربردهای این فن‌آوری در بخش کشاورزی، مطالعه حاضر با موضوع بررسی نگرش کارشناسان مدیریت جهاد کشاورزی شهرستان اصفهان پیرامون این کاربردها در سال 1391 انجام گردید. روش‌ها: مطالعه حاضر از نوع توصیفی- تحلیلی- همبستگی به روش مقطعی بود که به روش سرشماری بر روی کارشناسان مشغول به فعالیت در مرکز مدیریت جهاد کشاورزی شهرستان اصفهان انجام شد. برای جمع‌آوری داده‌ها از پرسش‌نامه محقق ساخته استفاده گردید که روایی و پایایی آن مورد تأیید قرار گرفت. اطلاعات به دست آمده با استفاده از ضریب همبستگی Pearson و آزمون‌های Mann-Whitney و Kruskal-wallis به وسیله نرم‌افزار SPSS نسخه 19 مورد تجزیه و تحلیل قرار گرفت. یافته‌ها: میانگین سنی کارشناسان 5/36 سال با انحراف معیار 4/9 بود. بین متغیرهای سن و سطح تحصیلات با نگرش کارشناسان رابطه معنی‌داری وجود نداشت؛ در صورتی که بین سابقه خدمت و تعداد دوره‌های آموزشی پیرامون فن‌آوری نانو که کارشناسان در آن‌ها شرکت نموده‌اند با نگرش آنان رابطه معنی‌دار و معکوس وجود داشت. بحث و نتیجه‌گیری: از دیدگاه کارشناسان، فن‌آوری نانو سبب تولید موادی با خواص بهتر، هزینه کمتر و دوام بیشتر می‌شود و همین طور فن‌آوری نانو رقیب سایر فن‌آوری‌ها نیست، بلکه مکمل و پایه آن‌ها است. نگرش کارشناسان در مواردی مانند "فن‌آوری نانو یک موج تکنولوژیکی گذرا و کم‌دوام به شمار می‌آید" و یا "فن‌آوری نانو و کاربردهای آن، بیشتر جنبه تخیلی و غیر واقعی دارد" منفی بود.
